# Supplementary material for: Genomic Sequencing and Characterization of Two Auricularia Species from the Qinling Region: Insights into Evolutionary Dynamics and Secondary Metabolite Potential
Source: J Fungi (Basel). 2025 May 20;11(5):395. doi: 10.3390/jof11050395 (PMC12113221; doi:10.3390/jof11050395)
Supplement: Supplementary file 1 [file jof-11-00395-s001.zip › Supplemental Material.pdf]

Genomic Sequencing and Characterization of Two *Auricularia* Species from the Qinling Region: Insights into Evolutionary Dynamics and Secondary Metabolite Potential

Jianzhao Qi [1,2,3,*], Shijie Kang [1], Ming Zhang [1], Shen Qi [1], Yulai Li [1], Khassanov Vadim [4], Shuangtian Du [2] and Minglei Li [2,3,*]

1 Shaanxi Key Laboratory of Natural Products & Chemical Biology, College of Chemistry & Pharmacy, Northwest A&F University, Yangling, Xianyang 712100, China
2 Center of Edible Fungi, Northwest A&F University, Yangling, Xianyang 712100, China
3 School of Soil and Water Conservation Science and Engineering, Northwest A&F University, Yangling, Xianyang 712100, China
4 Department of Plant Protection and Quarantine, Faculty of Agronomy, S. Seifullin Kazakh Agrotechnical University, Zhenis Avenue, Astana 010011, Kazakhstan
* Correspondence: qjz@nwafu.edu.cn (J.Q.); mlli@nwafu.edu.cn.com (M.L.)

Content

Table S1. Next-generation sequencing data of *Auricularia sp.* qinling M12 *and Auricularia sp.* qinling M13 genome. 1
Table S2. Estimation of genome size of *Auricularia* sp. qinling M12 *and Auricularia* sp. qinling M13. 2
Table S3. Statistical table of *Auricularia* sp. qinling M12 *and Auricularia* sp. qinling M13 assembly results. 3
Table S4. Statistical table of the length of the assembly sequence of *Auricularia* sp. qinling M12 *and Auricularia* sp. qinling M13. 4
Table S5. Statistics of BUSCO evaluation of *Auricularia* sp. qinling M12 *and Auricularia* sp. qinling M13 genome. 5
Table S6. Genetic Information the Statistical Table of protein-coding genes. 6
Table S7. Statistics of non-coding RNA annotation results in *Auricularia* sp. qinling M12 *and Auricularia* sp. qinling M13 genome....7
Table S8. Statistics of *Auricularia* sp. qinling M12 *and Auricularia* sp. qinling M13 repetitive sequence annotation results....8
Table S9. Anonation statistics table of *Auricularia* sp. qinling M12 *and Auricularia* sp. qinling M13....10
Table S10. Statistics for SNP of *Auricularia* sp. qinling M12 *and Auricularia* sp. qinling M13. 11
Table S11. The source (URL) statistics for 28 Basidiomycota and an Ascomycete used to phylogenetic analysis....13
Table S12. Statistics for CYP450 number and Percentage of 28 Basidiomycota and an Ascomycete. 15
Table S13. The composition of CAZymes of 29 representative Basidiomycetes. 16
Table S14. The source (URL) statistics for 29 representative Basidiomycetes used to CAZymes analysis. 17
Table S15. Statistics for SSR of seven *Auricularia* mushroom. 19
Table S16. Terpene enzymes in *Auricularia* sp. qinling M12 *and Auricularia* sp. qinling M13 genomes....21
Figure S1. Kmer-Depth and Kmer Species-Frequency Distribution Plot of *Auricularia* sp. qinling M12. 22
Figure S2. Kmer-Depth and Kmer Species-Frequency Distribution Plot of *Auricularia* sp. qinling M13. 24
Figure S3. Statistical map of functional annotation classification based on GO database of *Auricularia* sp. qinling M12....25
Figure S4. Statistical map of functional annotation classification based on GO database of *Auricularia* sp. qinling M13....26
Figure S5. Statistical Chart of COG Functional Annotated Classification of *Auricularia* sp. qinling M12. 27
Figure S6. Statistical Chart of COG Functional Annotated Classification of *Auricularia* sp. qinling M13. 28
Figure S7. KEGG Pathway Functional Classification Chart of *Auricularia* sp. qinling M12.

....29
Figure S8. KEGG Pathway Functional Classification Chart of *Auricularia* sp. qinling M13. 30
Figure S9. Domain annotation based on the Pfam database of *Auricularia* sp. qinling M12. 31
Figure S10. Domain annotation based on the Pfam database of *Auricularia* sp. qinling M13. 32
Figure S11. Ka comparisons of three *Auricularia* species. 33
Figure S12. Ks comparisons of three *Auricularia* species. 34
Figure S13. Evolutionary tree analysis of STSs. 35

Table S1. Next-generation sequencing data of *Auricularia sp.* qinling M12 *and Auricularia sp.* qinling M13 genome.

Item	Value	
	***Auricularia* sp. qinling M12**	***Auricularia* sp. qinling M13**
Insert size	113,063,776 bp	
Raw data	16,959,566,400 bp	5,825,114,700 bp
Clean data	16,957,605,300 bp	5,824,634,700 bp
Clean data Q20	97.65%	98.34%
Clean data Q30	93.22%	95.08%
Clean data GC	54.72%	55.02%

Table S2. Estimation of genome size of *Auricularia* sp. qinling M12 *and* *Auricularia* sp. qinling M13.

Item	Number	
	Auricularia sp. qinling M12	*Auricularia* sp. qinling M13
kmer	15	15
Genome size	55,197,415 bp	49,617,931 bp
Heterozygosity	2.579%	2.343%
Genome Repeat Length	25,068,819 bp	19,585,395 bp
Genome Unique Length	30,128,595 bp	30,032,536 bp

Genome size was estimated using genomescope v2.0 (http://genomescope.org/genomescope2.0/) software.

Table S3. Statistical table of *Auricularia* sp. qinling M12 *and Auricularia* sp. qinling M13 assembly results.

Item	Value	
	Auricularia sp. qinling M12	*Auricularia* sp. qinling M13
Total Length (bp)	56,039,961	52,096,326
N50 length (bp)	3,257,518	2,317,991
N90 length (bp)	1,088,702	1,233,194
GC Content(%)	57.01	57.09
N rate(%)	0	0
Total_number	38	26
Average(bp)	1,474,735.82	2,003,658.69
Median(bp)	979,030.50	1,971,398.50
Min(bp)	58,450	86,453
Max(bp)	7,410,467	4,551,741

Table S4. Statistical table of the length of the assembly sequence of *Auricularia* sp. qinling M12 *and Auricularia* sp. qinling M13.

Species	Chr	Length (bp)	Nrate	contig number
Auricularia sp. qinling M12	Chr1	4,590,200	0	3
	Chr2	4,985,111	0	2
	Chr3	3,866,365	0	2
	Chr4	3,304,998	0	1
	Chr5	3,746,208	0	2
	Chr6	4,267,849	0	1
	Chr7	3,879,286	0	2
	Chr8	9,151,808	0	3
	Chr9	3,658,999	0	1
	Chr10	4,428,527	0	4
	Chr11	2,451,749	0	1
	Chr12	5,261,384	0	6
	chrUnn	1,008,338	0	13
Auricularia sp. qinling M13	Ctg1	9,420,553	0	
	Ctg2	5,178,878	0	
	Ctg3	4,322,239	0	
	Ctg4	4,551,741	0	
	Ctg5	4,406,950	0	
	Ctg6	4,342,189	0	
	Ctg7	3,876,945	0	
	Ctg8	3,838,980	0	
	Ctg9	3,739,673	0	
	Ctg10	3,648,102	0	
	Ctg11	3,216,104	0	
	Ctg12	1,246,538	0	
	Ctg13	220,981	0	
	Ctg14	86,453	0	

Table S5. Statistics of BUSCO evaluation of *Auricularia* sp. qinling M12 *and* *Auricularia* sp. qinling M13 genome.

Item	*Auricularia* sp. qinling M12		*Auricularia* sp. qinling M13	
	**Number**	**Percent (%)**	**Number**	**Percent (%)**
Complete BUSCOs (C)	706	93.1	711	93.8
Complete and single-copy BUSCOs (S)	620	81.8	678	89.4
Complete and duplicated BUSCOs (D)	86	11.3	33	4.4
Fragmented BUSCOs (F)	9	1.2	6	0.8
Missing BUSCOs (M)	43	5.7	41	5.4
Total BUSCO groups searched (n)	758	100.0	758	100.0

Single-copy for single-copy BUSCOs; duplicated for multicopy BUSCOs; Fragmented for fragmented BUSCOs; Missing for missing BUSCOs. The predicted genes were assessed for completeness using the BUSCO software (version: 4.1.4) based on the fungi database (fungi_odb10).

Table S6. Genetic Information the Statistical Table of protein-coding genes.

Item	Number	
	***Auricularia* sp. qinling M12**	***Auricularia* sp. qinling M13**
Gene number	17,043	16,484
Average of mRNA length (bp)	1,704.25	1,868.94
Average of cds length (bp)	1,272.25	1,285.88
Average of exon length (bp)	209.01	195.02
Average of exon number	6.09	6.59
Average of intron length (bp)	84.91	104.07
Total number of exon	103,741	108,687
Total number of intron	86,698	92,203
Total intron length	7,361,104	9,595,461

Table S7. Statistics of non-coding RNA annotation results in *Auricularia* sp. qinling M12 *and Auricularia* sp. qinling M13 genome.

Sample	Class	number	Total Len(bp)	Mean Len(bp)
Auricularia sp. qinling M12	rRNA	64	111,533	1,742
	sRNA	1	296	296
	snRNA	23	3,167	137
	tRNA	115	9,469	82
Auricularia sp. qinling M13	rRNA	39	68,116	1,746
	sRNA	1	296	296
	snRNA	23	3,179	138
	tRNA	110	9,057	82

rRNA is ribosomal RNA; tRNA is transport RNA; sRNA is small regulatory RNA; snRNA is nucleolar small RNA. **totalLen** and **meanLen** are the total length and mean length.

Table S8. Statistics of *Auricularia* sp. qinling M12 *and Auricularia* sp. qinling M13 repetitive sequence annotation results.

Species	Type	number	Total Len(bp)	Coverage/%	Subfamily
Auricularia sp. qinling M12	LTR	1,444	1,479,390	2.71	
	LTR	1,012	1,108,852	2.03	Gypsy
	LTR	269	245,352	0.45	Copia
	DNA	661	317,692	0.58	
	LINE	714	588,344	1.08%	
	SINE	9	799	0.00	
	Satellite	26	2,555	0.00	
	Simple repeat	9,547	436,035	0.80	
	Low complexity	791	41,454	0.08	
	Other	87	20,844	0.04	
	Unknown	8,563	4,884,295	8.95	
	Total	21,842	7,715,804	14.13	
Auricularia sp. qinling M13	LTR	1,513	1,847,445	3.55	
	LTR	1,194	1,587,984	3.05	Gypsy
	LTR	238	224,622	0.43	Copia
	DNA	682	377,179	0.72	
	LINE	525	346,301	0.66	
	SINE	1	82	0.00	

Satellite	26	2,267	0.00
Simple repeat	8,838	403,238	0.77
Low complexity	766	39,815	0.08
Other	85	119,353	0.23
Unknown	7,840	4,127,439	7.92
Total	20,276	7,237,846	13.89

Table S9. Anonation statistics table of *Auricularia* sp. qinling M12 *and* *Auricularia* sp. qinling M13.

Species	**Item**	**Count**	Percentage/%
Auricularia sp. qinling M12	All	17,043	100.00
	Annotation	15,425	90.51
	Uniprot	6,738	39.54
	Pfam	10,636	62.41
	Refseq	5,338	31.32
	Nr	15,235	89.39
	Interproscan	10,653	62.51
	GO	6,668	39.12
	KEGG	4,733	27.77
	Pathway	2,903	17.03
	COG	1,478	8.67
Auricularia sp. qinling M13	All	16,484	100.00
	Annotation	14,597	88.55
	Uniprot	6,348	38.51
	Pfam	10,305	62.52
	Refseq	5,074	30.78
	Nr	14,394	87.32
	Interpro	10,267	62.28
	GO	6,284	38.12
	KEGG	4,498	27.29
	Pathway	2,714	16.46
	KOG	1,394	8.46

Table S10. Statistics for SNP of *Auricularia* sp. qinling M12 *and Auricularia* sp. qinling M13.

Species	No.	Number
Auricularia QingLing heimuer M12	Chr1	35,813
	Chr2	67,718
	Chr3	60,084
	Chr4	46,569
	Chr5	62,193
	Chr6	56,016
	Chr7	40,255
	Chr8	134,679
	Chr9	56,080
	Chr10	58,411
	Chr11	31,307
	Chr12	11,149
	Ctg1	116
	Ctg2	131
	Ctg3	0
	Ctg4	23
	Ctg5	44
	Ctg6	315
	Ctg7	475
	Ctg8	203
	Ctg9	194
	Ctg10	402
	Ctg11	13
	Ctg12	241
	Ctg13	0
	Total	662,431
Auricularia QingLing heimuer M13	Ctg1	120,699
	Ctg2	60,041
	Ctg3	59,121
	Ctg4	43,087
	Ctg5	36,853
	Ctg6	53,372
	Ctg7	55,502
	Ctg8	44,559
	Ctg9	42,706
	Ctg10	38,294
	Ctg11	30,942

	Ctg12	3,602
	Ctg13	1,072
	Ctg14	188
	Total	590,038

Table S11. The source (URL) statistics for 28 Basidiomycota and an Ascomycete used to phylogenetic analysis.

Species	Source
Auricularia sp. qinling M12	This study
Auricularia sp. qinling M13	This study
Auricularia subglabra TFB-10046 SS5	https://www.ncbi.nlm.nih.gov/datasets/genome/GCF_000265015.1/
Elmerina caryae	https://mycocosm.jgi.doe.gov/Elmca1/Elmca1.home.html
Exidia glandulosa	https://www.ncbi.nlm.nih.gov/datasets/genome/GCA_001632375.1/
Oliveonia pauxilla	https://mycocosm.jgi.doe.gov/Olipa1/Olipa1.home.html
Porpomyces mucidus	https://mycocosm.jgi.doe.gov/Pormuc1/Pormuc1.home.html
Sebacina vermifera	https://mycocosm.jgi.doe.gov/Sebve1/Sebve1.home.html
Hydnum rufescens	https://mycocosm.jgi.doe.gov/Hydru2/Hydru2.home.html
Sistotrema raduloides	https://mycocosm.jgi.doe.gov/Sisrad1/Sisrad1.home.html
Cantharellus anzutake	https://mycocosm.jgi.doe.gov/Cananz1/Cananz1.home.html
Sistotrema brinkmannii	https://mycocosm.jgi.doe.gov/Sisbri1/Sisbri1.home.html
Tulasnella calospora	https://mycocosm.jgi.doe.gov/Tulca1/Tulca1.home.html
Tulsanella inquilina	
Rhizoctonia solani	https://www.ncbi.nlm.nih.gov/datasets/genome/GCF_016906535.1/
Thanatephorus cucumeris	https://mycocosm.jgi.doe.gov/Thacu1/Thacu1.home.html
Calocera	https://mycocosm.jgi.doe.gov/Calco1/Calco1.home.html

cornea	
Dacryopinax primogenitus	https://mycocosm.jgi.doe.gov/Dacsp1/Dacsp1.home.html
Trichosporon oleaginosus	https://mycocosm.jgi.doe.gov/Triol1/Triol1.home.html
Trichosporon asahii	https://mycocosm.jgi.doe.gov/Trias8904/Trias8904.home.html
Tremella mesenterica	https://mycocosm.jgi.doe.gov/Treme1/Treme1.home.html
Dioszegia hungarica	https://mycocosm.jgi.doe.gov/Diohu1/Diohu1.home.html
Filobasidium floriforme	https://mycocosm.jgi.doe.gov/Filflo1/Filflo1.home.html
Puccinia striiformis	https://mycocosm.jgi.doe.gov/Pucstr1/Pucstr1.home.html
Naohidea sebacea	https://genome.jgi.doe.gov/portal/NaosebStandDraft_FD/NaosebStandDraft_FD.info.html
Rhodotorula graminis	https://mycocosm.jgi.doe.gov/Rhoba1/Rhoba1.home.html
Acaromyces ingoldii	https://mycocosm.jgi.doe.gov/Acain1/Acain1.home.html
Ustilago maydis	https://mycocosm.jgi.doe.gov/Ustma2_2/Ustma2_2.home.html
Aspergillus oryzae	https://mycocosm.jgi.doe.gov/Aspor1/Aspor1.home.html

Table S12. Statistics for CYP450 number and Percentage of 28 Basidiomycota and an Ascomycete.

Species	Number	Percentage (%)
Auricularia sp. qinling M12	150	63.03
Auricularia sp. qinling M13	153	64.29
Auricularia subglabra TFB-10046 SS5	238	100.00
Elmerina caryae	143	60.08
Exidia glandulosa	196	82.35
Oliveonia pauxilla	94	39.50
Porpomyces mucidus	101	42.44
Sebacina vermifera	131	55.04
Hydnum rufescens	47	19.75
Sistotrema raduloides	102	42.86
Cantharellus anzutake	63	26.47
Sistotrema brinkmannii	47	19.75
Tulasnella calospora	140	58.82
Tulsanella inquilina	141	59.24
Rhizoctonia solani	197	82.77
Thanatephorus cucumeris	195	81.93
Calocera cornea	130	54.62
Dacryopinax primogenitus	123	51.68
Trichosporon oleaginosus	16	6.72
Trichosporon asahii	19	7.98
Tremella mesenterica	8	3.36
Dioszegia hungarica	16	6.72
Filobasidium floriforme	11	4.62
Puccinia striiformis	26	10.92
Naohidea sebacea	21	8.82
Rhodotorula graminis	11	4.62
Acaromyces ingoldii	37	15.55
Ustilago maydis	20	8.40
Aspergillus oryzae	155	65.13

Table S13. The composition of CAZymes of 29 representative Basidiomycetes.

Species	AA	CBM	CE	GH	GT	PL
Agaricus bitorquis BH01	22	9	14	66	4	8
Agaricus bisporus var *bisporus* H97	38	10	20	80	6	8
Armillaria mellea ELDO17	48	11	25	90	1	15
Auricularia Qinglingheimuer M12	80	14	41	216	2	22
Auricularia Qinglingheimuer M13	83	16	35	212	1	24
Boletus edulis BED1	19	1	7	74	3	1
Cordyceps militaris CM01	22	3	6	71	3	3
Cyclocybe aegerita AAE3	63	11	17	89	2	10
Grifola frondosa 9006-11	37	2	5	51	2	3
Hericium alpestre NPCB A08	32	5	11	78	3	4
Hericium coralloides FP-101451	34	5	16	87	1	3
Hericium erinaceus CS_4	42	5	15	80	4	4
Lactarius deliciosus EDB83	47	3	7	64	6	5
Lentinula edodes Lenedo1	40	10	15	131	2	8
Lyophyllum decaste LRG-d1-5	64	17	21	114	5	17
Morchella snyderi CBS 144464	43	5	19	91	3	21
Oudemansiella raphanipes CGG-A-s2	89	7	26	153	8	21
Paxillus involutus ATCC 200175	29	3	8	63	4	5
Pleurotus ostreatus PC9	83	20	19	121	2	20
Pleurotus giganteus zhudugu2	81	15	14	107	4	11
Sparassis crispa SCP 1.1	15	0	4	58	3	5
Stropharia rugosoannulata A15	80	11	29	105	2	6
Tremella mesenterica Fries	4	1	2	19	2	2
Tuber melanosporum Mel28	18	1	5	32	2	2

Table S14. The source (URL) statistics for 29 representative Basidiomycetes used to CAZymes analysis.

Species	Source
Agaricus bitorquis BH01	https://www.ncbi.nlm.nih.gov/datasets/genome/GCA_030246685.1/
Agaricus bisporus var *bisporus* H97	https://www.ncbi.nlm.nih.gov/datasets/genome/GCF_000300575.1/
Armillaria mellea ELDO17	https://www.ncbi.nlm.nih.gov/datasets/genome/GCA_030407055.1/
Auricularia Qinglingheimuer M12	This study
Auricularia Qinglingheimuer M13	This study
Boletus edulis BED1	https://www.ncbi.nlm.nih.gov/datasets/genome/GCA_015179015.1/
Cordyceps militaris CM01	https://www.ncbi.nlm.nih.gov/datasets/genome/GCF_000225605.1/
Cyclocybe aegerita AAE3	https://www.ncbi.nlm.nih.gov/datasets/genome/GCA_902728275.1/
Grifola frondosa 9006-11	https://www.ncbi.nlm.nih.gov/datasets/genome/GCA_001683735.1/
Hericium alpestre NPCB A08	
Hericium coralloides FP-101451	https://mycocosm.jgi.doe.gov/Hercor1/Hercor1.home.html
Hericium erinaceus CS_4	https://www.ncbi.nlm.nih.gov/datasets/genome/GCA_006506795.2/
Lactarius deliciosus EDB83	
Lentinula edodes Lenedo1	https://www.ncbi.nlm.nih.gov/datasets/genome/GCF_021015755.1/
Lyophyllum decaste LRG-d1-5	https://www.ncbi.nlm.nih.gov/datasets/genome/GCA_026258425.1/
Morchella snyderi CBS 144464	https://www.ncbi.nlm.nih.gov/datasets/genome/GCA_024521645.1/
Oudemansiella raphanipes CGG-A-s2	https://www.ncbi.nlm.nih.gov/datasets/genome/GCA_036872995.1/
Paxillus involutus	https://www.ncbi.nlm.nih.gov/datasets/genome/GCA_000827475.1/

ATCC 200175	
Pleurotus ostreatus PC9	https://mycocosm.jgi.doe.gov/PleosPC9_1/PleosPC9_1.home.html
Pleurotus giganteus zhudugu2	https://www.ncbi.nlm.nih.gov/datasets/genome/GCA_036873075.1/
Sparassis crispa SCP 1.1	https://www.ncbi.nlm.nih.gov/datasets/genome/GCF_003851025.1/
Stropharia rugosoannulata A15	https://www.ncbi.nlm.nih.gov/datasets/genome/GCA_036873085.1/
Tremella mesenterica Fries	https://www.ncbi.nlm.nih.gov/datasets/genome/GCF_000271645.1/
Tuber melanosporum Mel28	https://www.ncbi.nlm.nih.gov/datasets/genome/GCF_000151645.1/

Table S15. Statistics for SSR of seven *Auricularia* mushroom.

Species	Motif	No.	Relative abundance (per/Mb)	Percentage (%)	Longest pattern
Auricularia auricula-judae B14-8	Monomer	740	18	24.92	$(A)_{91}$
	Dimer	610	15	20.54	$(AT)_{15}$
	Trimer	1395	33	46.97	$(GTA)_{16}$
	Tetramer	120	3	4.04	$(TACC)_{13}$
	Pentamer	80	2	2.69	$(GTTA)_{52}$
	Hexamer	25	1	0.84	$(GAAGA)_{16}$
	all SSRs	2970	71	100.00	$(GTTA)_{52}$
Auricularia cornea CCMJ2827	Monomer	1272	17	22.34	$(A)_{94}$
	Dimer	1770	23	31.09	$(GA)_{42}$
	Trimer	2304	30	40.47	$(GGC)_{22}$
	Tetramer	143	2	2.51	$(TCTG)_{11}$
	Pentamer	122	2	2.14	$(ACCTA)_{46}$
	Hexamer	82	1	1.44	$(GCGACT)_{12}$
	all SSRs	5693	75	100.00	$(ACCTA)_{46}$
Auricularia heimuer Dai 13782	Monomer	1058	22	28.37	$(T)_{102}$
	Dimer	766	16	20.54	$(AC)_{39}$
	Trimer	1616	34	43.34	$(CTA)_{39}$
	Tetramer	121	3	3.24	$(TTAT)_{12}$
	Pentamer	117	2	3.14	$(AGGTT)_{29}$
	Hexamer	51	1	1.37	$(TATTAG)_{15}$
	all SSRs	3729	78	100.00	$(AGGTT)_{29}$
Auricularia polytricha MG66	Monomer	607	16	48.25	$(T)_{46}$
	Dimer	176	5	13.99	$(TG)_{42}$
	Trimer	420	11	33.39	$(TTG)_{39}$
	Tetramer	25	1	1.99	$(TAAG)_{8}$
	Pentamer	4	0	0.32	$(TTATC)_{16}$
	Hexamer	26	1	2.07	$(TCTTTG)_{23}$
	all SSRs	1258	33	100.00	$(TCTTTG)_{23}$
Auricularia QingLing heimuer M12	Monomer	1021	19	24.19	$(T)_{67}$
	Dimer	929	17	22.01	$(CA)_{24}$
	Trimer	1903	35	45.09	$(AGT)_{36}$
	Tetramer	172	3	4.08	$(CTTT)_{10}$
	Pentamer	132	2	3.13	$(GTTAG)_{15}$
	Hexamer	63	1	1.49	$(CTCTCA)_{12}$
	all SSRs	4220	77	100.00	$(AGT)_{36}$
Auricularia QingLing	Monomer	903	18	24.05	$(A)_{49}$
	Dimer	862	17	22.96	$(AC)_{41}$

heimuer M13	Trimer	1693	34	45.09	$(AGT)_{22}$
	Tetramer	136	3	3.62	$(ACAG)_{9}$
	Pentamer	127	3	3.38	$(GGTTA)_{50}$
	Hexamer	34	1	0.91	$(GCAGGC)_{9}$
	all SSRs	3755	75	100.00	$(GGTTA)_{50}$
Auricularia subglabra TFB-10046	Monomer	1168	16	25.60	$(T)_{42}$
	Dimer	1264	17	27.70	$(GA)_{49}$
	Trimer	1740	24	38.13	$(TAC)_{31}$
	Tetramer	178	2	3.90	$(AGTA)_{20}$
	Pentamer	155	2	3.40	$(AACCT)_{31}$
	Hexamer	58	1	1.27	$(CCGTCC)_{11}$
	all SSRs	4563	63	100.00	$(AACCT)_{31}$

Table S16. Terpene enzymes in *Auricularia* sp. qinling M12 *and Auricularia* sp. qinling M13 genomes.

Gene ID	Anotation	Identities	Source	Accession number
M12_g15828.t1	sesquiterpene synthase	37.41%	*Hypholoma fasciculare*	UPX76569.1
M12_g13324.t1	sesquiterpene synthase	53.29%	*Auriscalpium vulgare*	BDI63091.1
M12_g3115.t1	sesquiterpene synthase	34.01%	*Hypholoma fasciculare*	UPX76569.1
M12_g9910.t1	hypothetical protein	73.54%	*Auricularia subglabra*	EJD40407.1
M12_g959.t1	farnesyl-diphosphate farnesyltransferase	83.20%	*Auricularia subglabra*	EJD41437.1
M12_g3769.t1	terpene synthase	87.15%	*Auricularia subglabra*	EJD41513.1
M13_g12586.t1	terpenoid synthase	48.23%	*Auricularia subglabra*	EJD48009.1
M13_g12833.t1	isoprenoid synthase	64.14%	*Auriculariales sp.*	KAH7101157.1
M13_g9589.t1	terpenoid synthase	70.35%	*Auricularia subglabra*	EJD53398.1
M13_g6529.t1	terpenoid synthase	67.66%	*Auricularia subglabra*	EJD35445.1
M13_g6776.t1	terpenoid synthase	87.78%	*Auricularia subglabra*	EJD40956.1
M13_g3626.t1	terpenoid synthase	57.72%	*Auricularia subglabra*	EJD44865.1
M13_g8992.t1	terpenoid synthase	64.94%	*Auriculariales sp.*	KAH7101707.1
M13_g1577.t1	farnesyl-diphosphate farnesyltransferase	83.40%	*Auricularia subglabra*	EJD41437.1
M13_g12252.t1	terpenoid synthase	70.97%	*Auricularia subglabra*	EJD44865.1

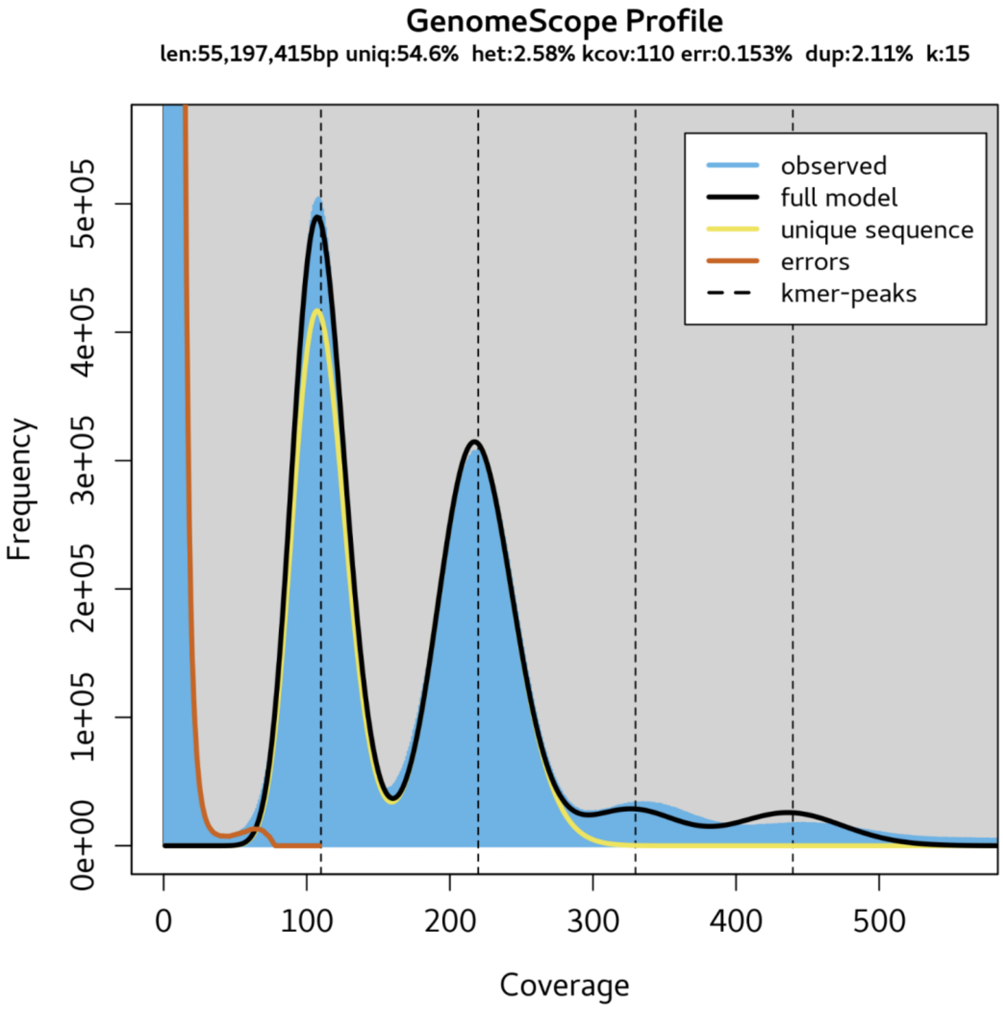

Figure S1. Kmer-Depth and Kmer Species-Frequency Distribution Plot of *Auricularia* sp. qinling M12.

The blue line represents the actual K-mer curve, the black line is the k-mer curve estimated by the model, the yellow line is the K-mer curve corresponding to the unique data, the red line represents the error curve due to sequencing errors, and the dashed line represents speculation K-mer peak.

Using the reads obtained by sequencing, K-mer-based analysis was used to estimate the genome size and heterozygosity. A K-mer refers to a sequence of K bp in length. Iteratively select a sequence of length K bases from a continuous sequence. If the length of the sequence is L and the length of the K-mer is K, then L-K+1 K-mers can be obtained. We take K-mers for the reads obtained by sequencing, and then count the frequency of each K-mer. According to the Lander waterman algorithm, the genome size (G) satisfies the following formula:

$$C_{base} = C_{k-mer} \times \frac{L}{L-K+1}$$

$$G = \frac{n_{k-mer}}{C_{k-mer}} = \frac{n_{base}}{C_{base}}$$

C_{base} and $C_{k\text{-}mer}$ are the expected depth of coverage and K-mer, and n base and n k-mer are the total number of bases and the total number of K-mers in the sequence. In the case of a certain amount of data, the depth frequency of K-mer is subject to Poisson distribution, so the peak of the K-mer depth

frequency distribution is the corresponding depth, which is used as an estimate of the expected depth of K-mer.

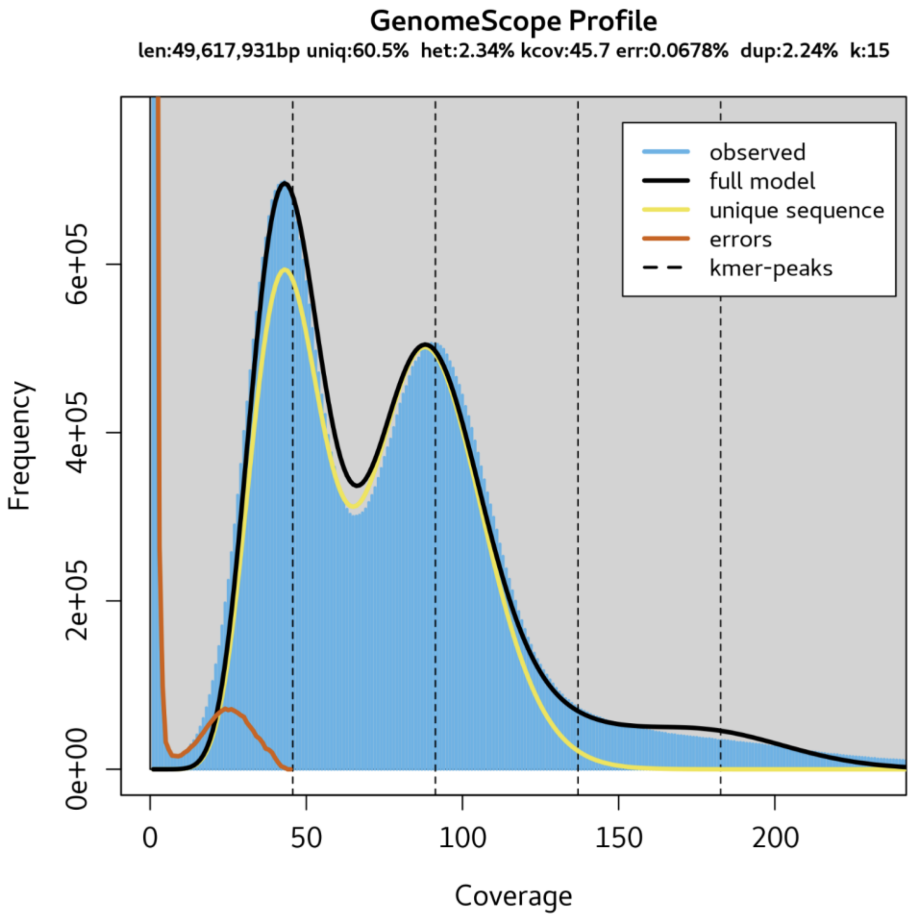

Figure S2. Kmer-Depth and Kmer Species-Frequency Distribution Plot of *Auricularia* sp. qinling M13.

The blue line represents the actual K-mer curve, the black line is the k-mer curve estimated by the model, the yellow line is the K-mer curve corresponding to the unique data, the red line represents the error curve due to sequencing errors, and the dashed line represents speculation K-mer peak.

Using the reads obtained by sequencing, K-mer-based analysis was used to estimate the genome size and heterozygosity. A K-mer refers to a sequence of K bp in length. Iteratively select a sequence of length K bases from a continuous sequence. If the length of the sequence is L and the length of the K-mer is K, then L-K+1 K-mers can be obtained. We take K-mers for the reads obtained by sequencing, and then count the frequency of each K-mer. According to the Lander waterman algorithm, the genome size (G) satisfies the following formula:

$$C_{base} = C_{k-mer} \times \frac{L}{L-K+1}$$

$$G = \frac{n_{k-mer}}{C_{k-mer}} = \frac{n_{base}}{C_{base}}$$

C_{base} and $C_{k\text{-}mer}$ are the expected depth of coverage and K-mer, and n base and n k-mer are the total number of bases and the total number of K-mers in the sequence. In the case of a certain amount of data, the depth frequency of K-mer is subject to Poisson distribution, so the peak of the K-mer depth frequency distribution is the corresponding depth, which is used as an estimate of the expected depth of K-mer.

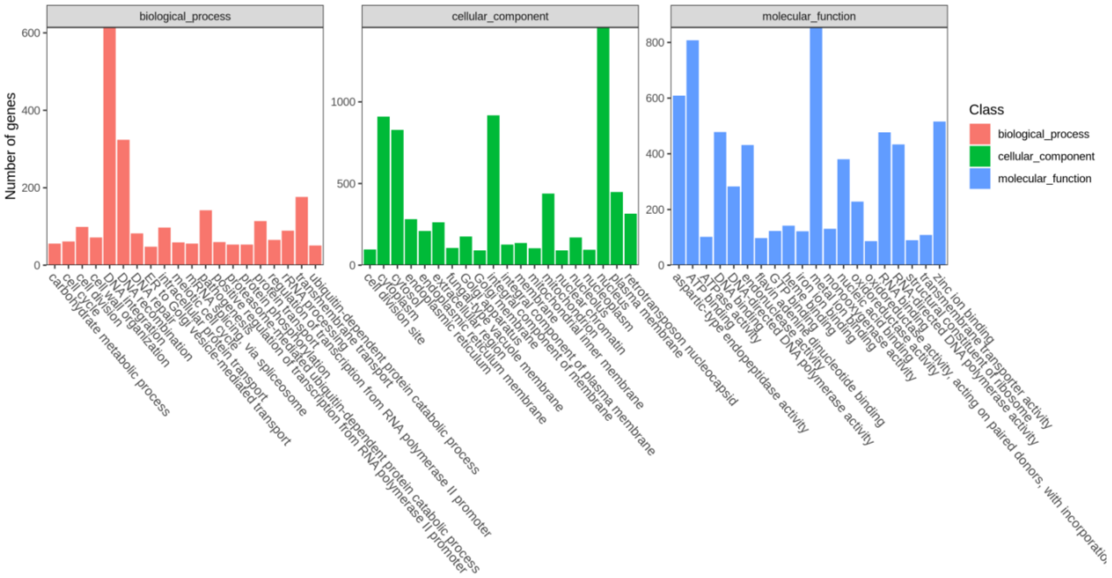

Figure S3. Statistical map of functional annotation classification based on GO database of *Auricularia* sp. qinling M12.

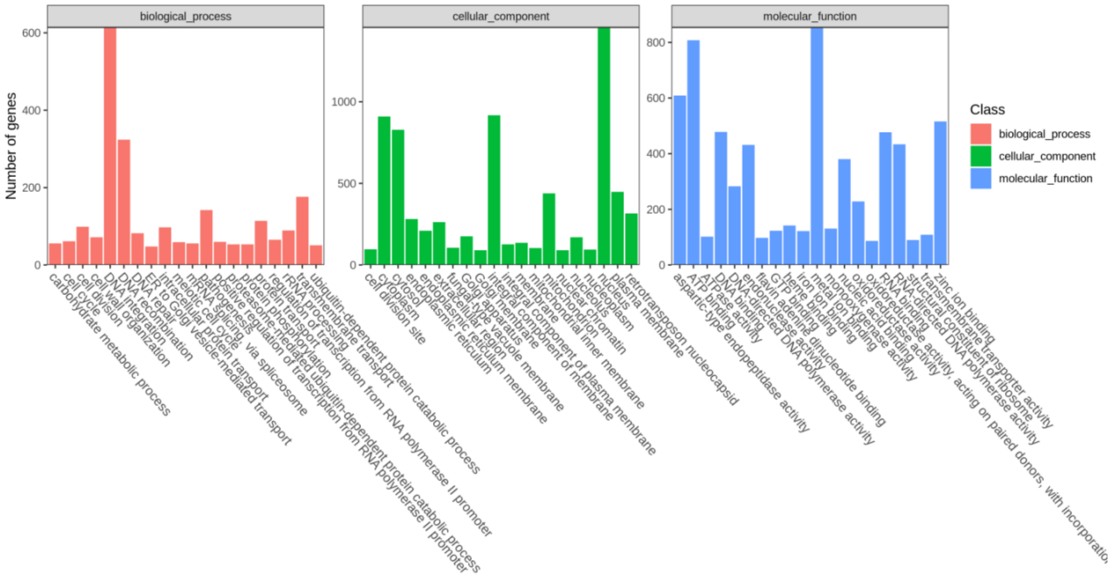

Figure S4. Statistical map of functional annotation classification based on GO database of *Auricularia* sp. qinling M13.

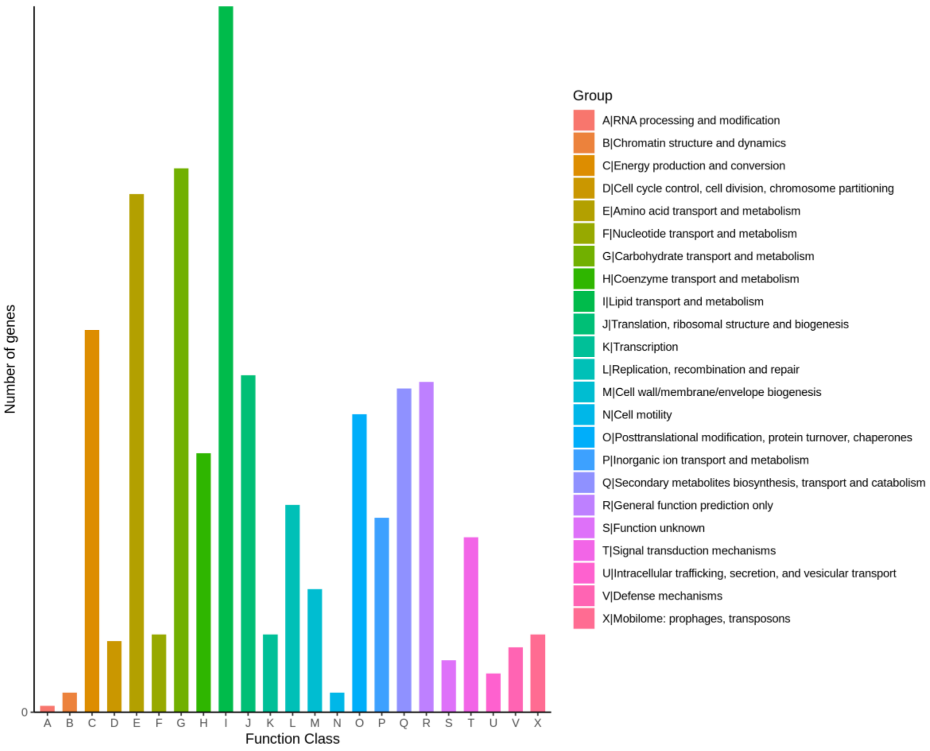

Figure S5. Statistical Chart of COG Functional Annotated Classification of *Auricularia* sp. qinling M12.

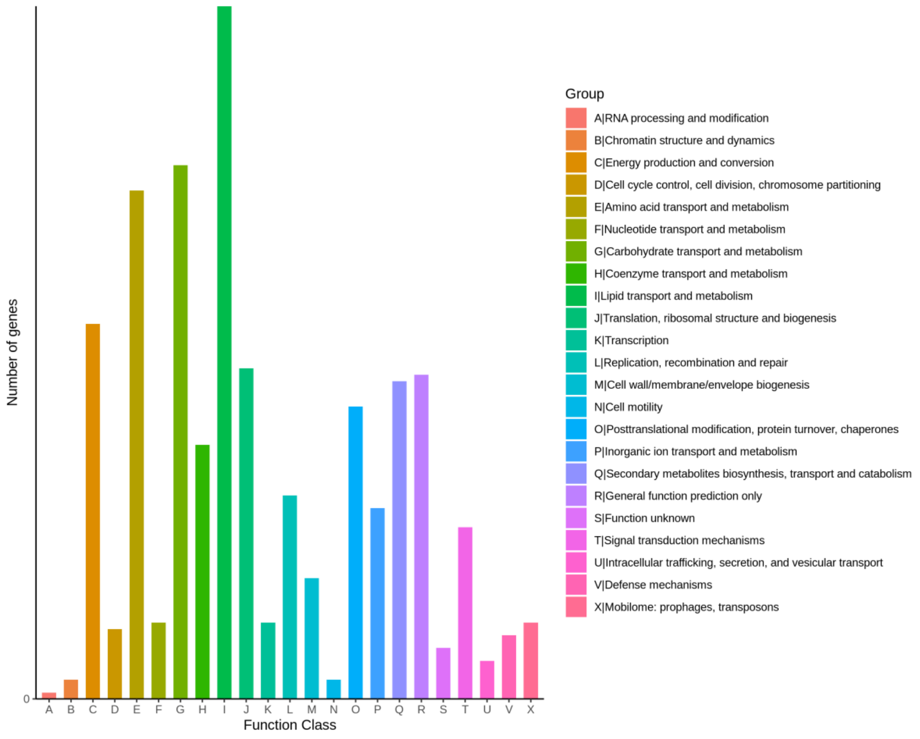

Figure S6. Statistical Chart of COG Functional Annotated Classification of *Auricularia* sp. qinling M13.

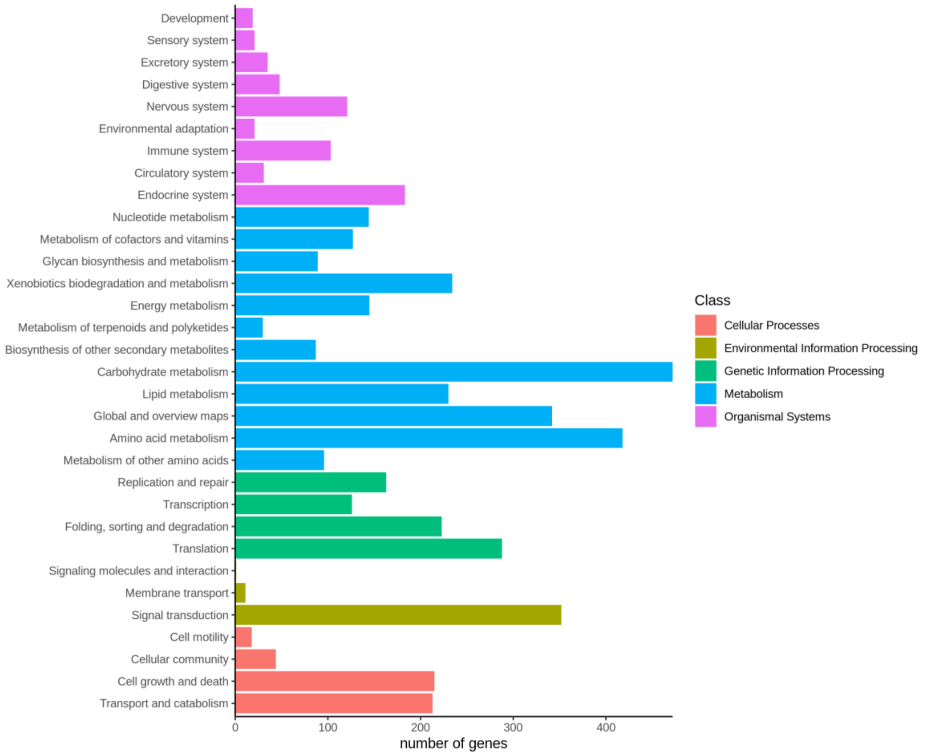

Figure S7. KEGG Pathway Functional Classification Chart of *Auricularia* sp. qinling M12.

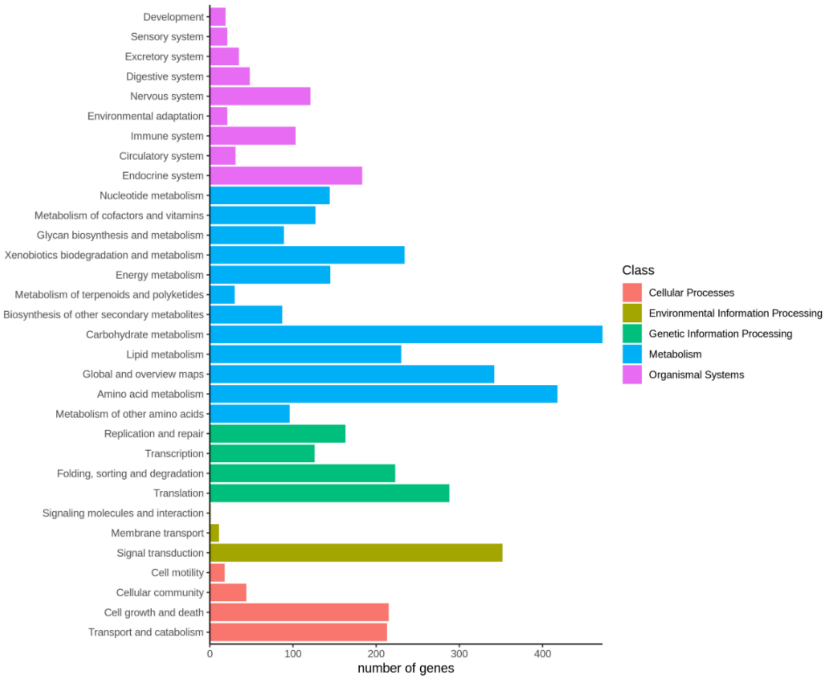

Figure S8. KEGG Pathway Functional Classification Chart of *Auricularia* sp. qinling M13.

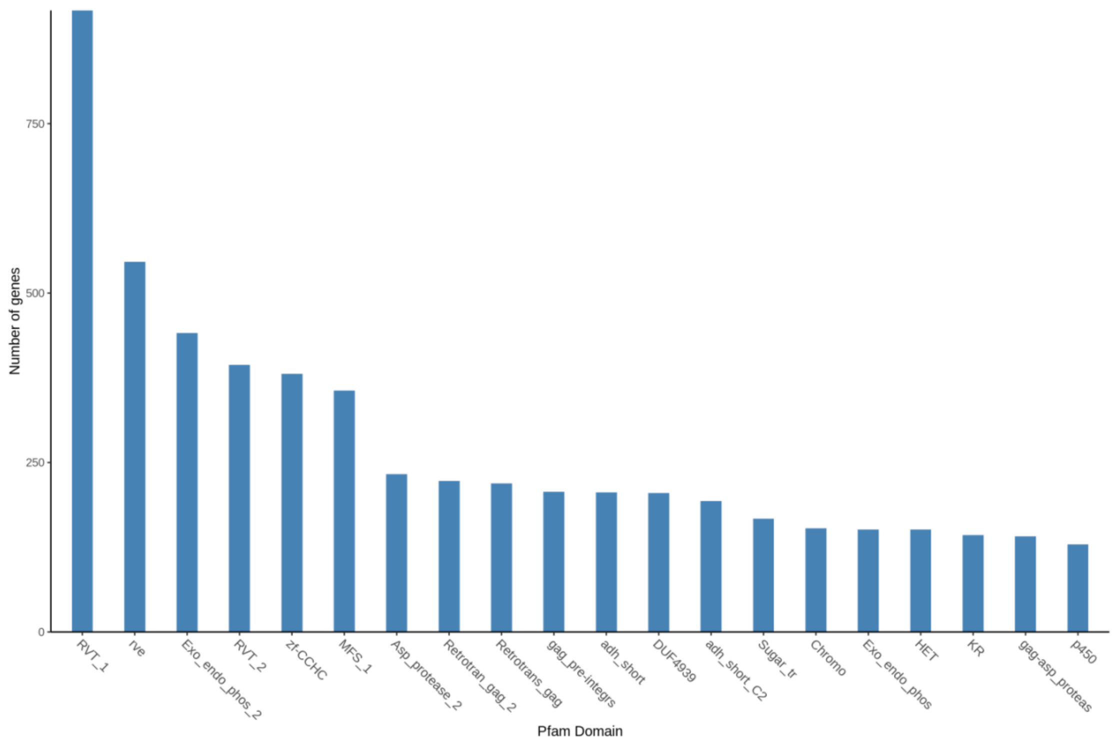

Figure S9. Domain annotation based on the Pfam database of *Auricularia* sp. qinling M12.

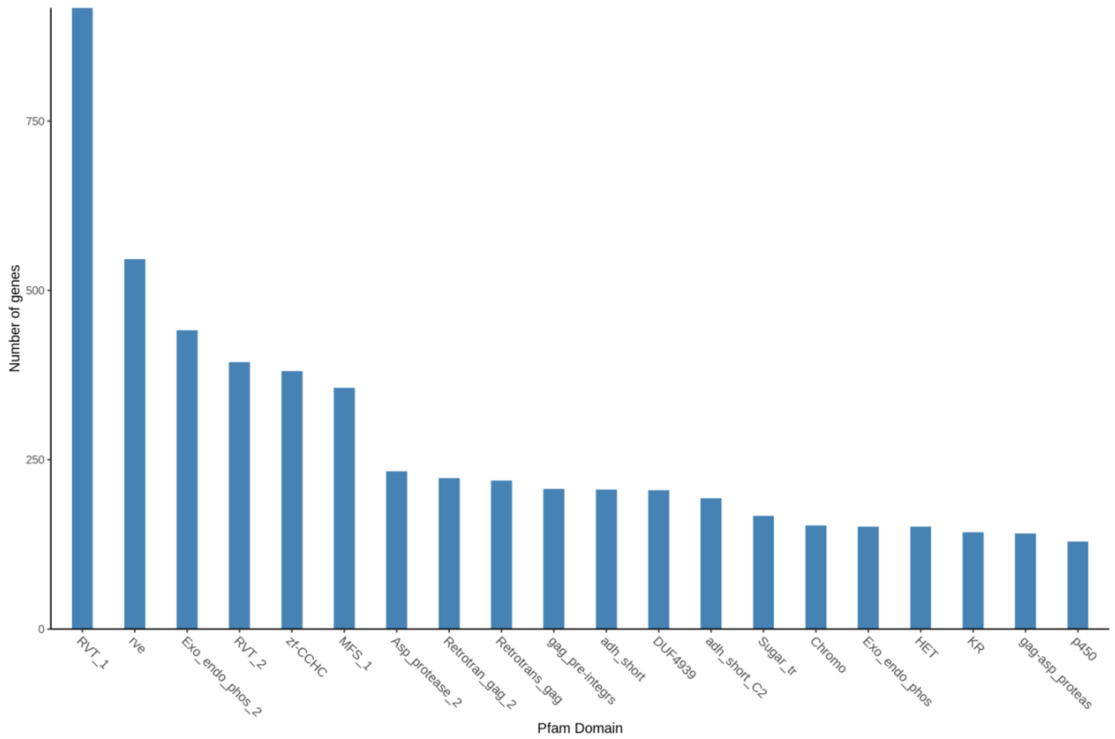

Figure S10. Domain annotation based on the Pfam database of *Auricularia* sp. qinling M13.

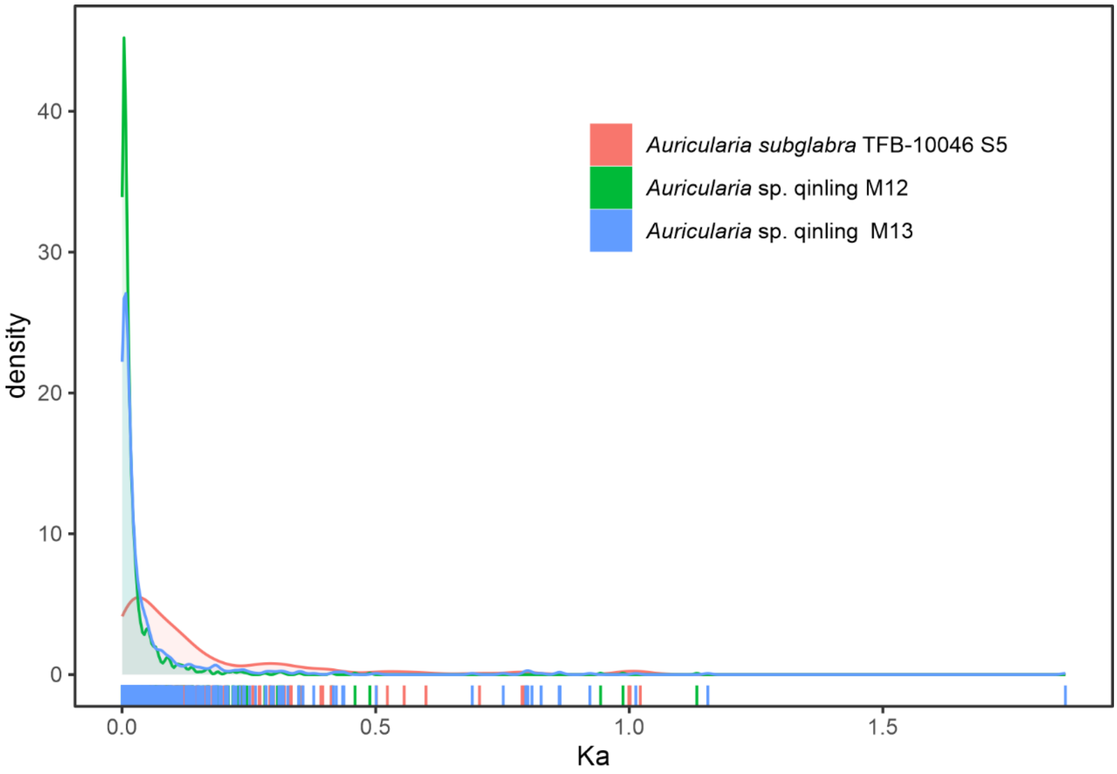

Figure S11. Ka comparisons of three *Auricularia* species.

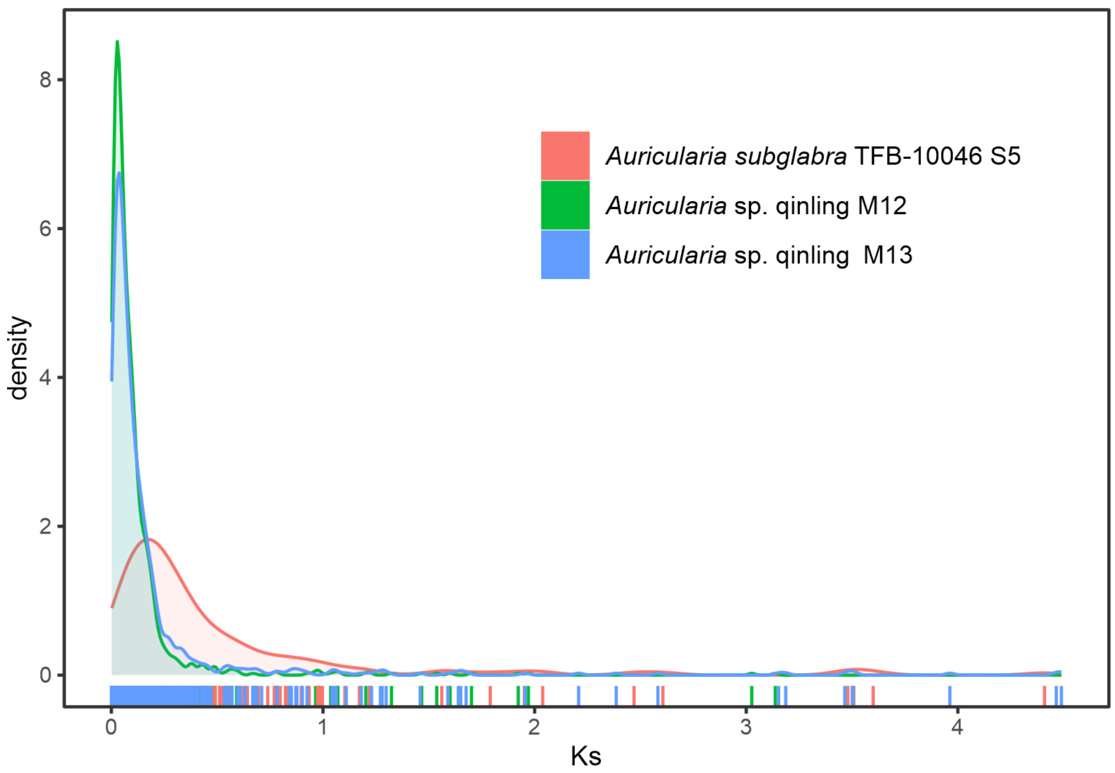

Figure S12. Ks comparisons of three *Auricularia* species.

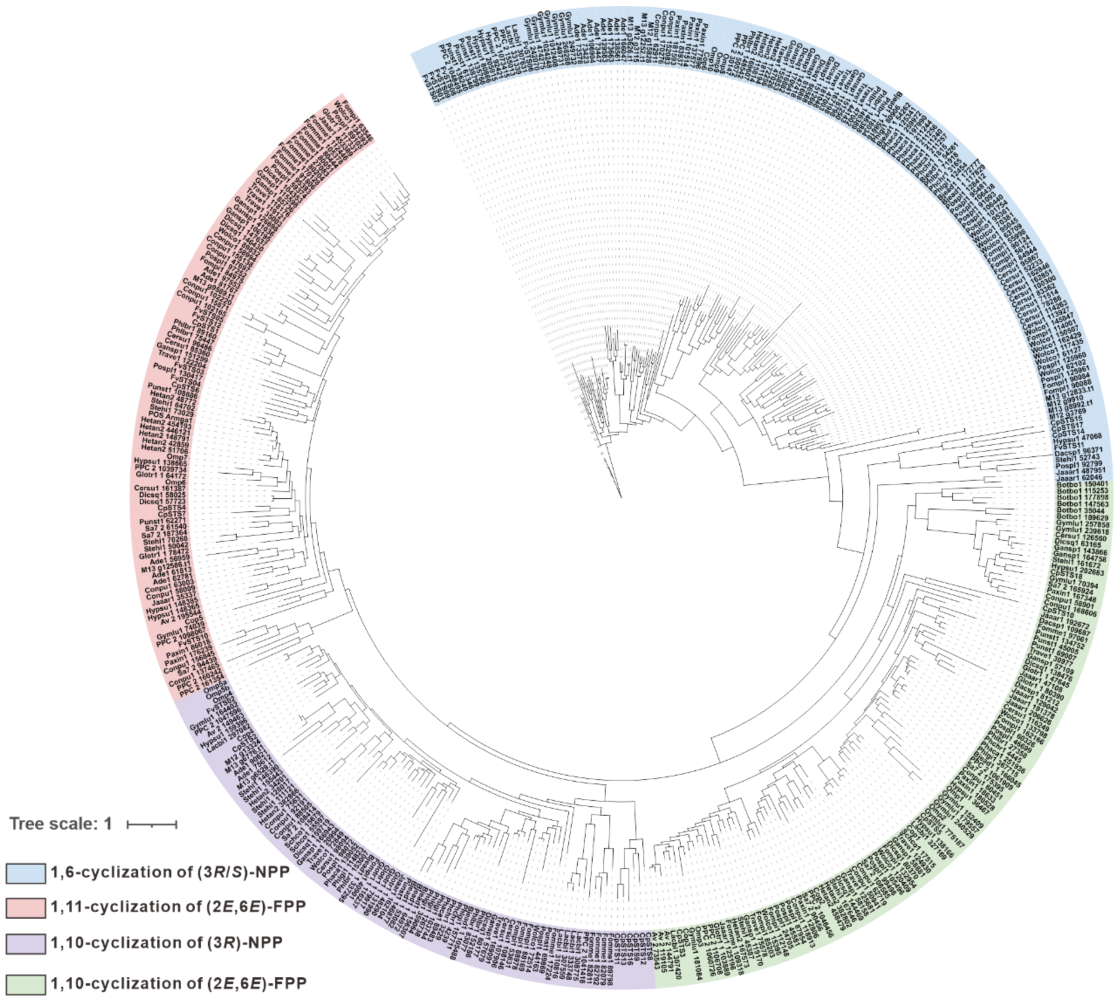

Figure S13. Evolutionary tree analysis of STSs.
